# Fully Biodegradable Elastomer-Based Device for Oral Macromolecule Delivery

**DOI:** 10.1021/acsabm.4c00147

**Published:** 2024-05-16

**Authors:** Reece McCabe, Lasse Højlund Eklund Thamdrup, Mahdi Ghavami, Anja Boisen

**Affiliations:** The Danish National Research Foundation and Villum Foundation’s Center for Intelligent Drug Delivery and Sensing Using Microcontainers and Nanomechanics (IDUN), Department of Health Technology, Technical University of Denmark, 2800 Kgs Lyngby, Denmark

**Keywords:** oral drug delivery, macromolecules, polymer, biodegradable elastomer, oral device

## Abstract

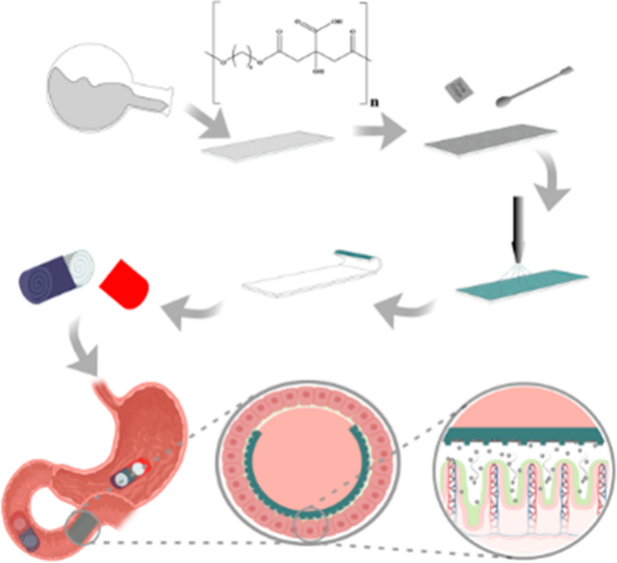

Oral devices, such as foil-type devices, show great potential for the delivery of poorly permeable macromolecules by enabling unidirectional release of the loaded pharmaceutical composition in close proximity to the epithelium in the small intestine or colon. However, one of the primary concerns associated with the use of foil-type devices so far has been the utilization of nonbiodegradable elastomers in the fabrication of the devices. Therefore, research into biodegradable substitute materials with similar characteristics enables drug delivery in a sustainable and environmentally friendly manner. In this study, a biodegradable elastomer, polyoctanediol citrate (POC), was synthesized via a one-pot reaction, with subsequent purification and microscale pattern replication via casting. The microstructure geometry was designed to enable fabrication of foil-type devices with the selected elastomer, which has a high intrinsic surface free energy. The final elastomer was demonstrated to have an elastic modulus ranging up to 2.2 ± 0.1 MPa, with strain at failure up to 110.1 ± 1.5%. Devices were loaded with acetaminophen and enterically coated, demonstrating 100% release at 2.5 h, following dissolution for 1 h in 0.1 M hydrochloric acid and 1.5 h in pH 6.8 phosphate-buffered saline. The elastomer demonstrated promising properties based on mechanical testing, surface free energy evaluation, and degradation studies.

## Introduction

Recent developments in the field of oral devices have shown an ability to directly interface the epithelium in the stomach, small intestine, and colon, which can offer the opportunity to deliver clinically relevant doses of, e.g., macromolecules with poor permeability.^1^ These devices seek to improve the absorption of active pharmaceutical ingredients (API) that are typically administered intravenously or subcutaneously, such as peptides and proteins. Such macromolecular compositions are of clinical and commercial interest, as they offer higher biological specificity and present fewer side effects than conventional small molecular drugs. They are typically water-soluble, yet due to high molecular weight, they are poorly permeable.^2^ Various oral device concepts have been reported, including direct injection of solid or liquid peptide/protein formulations into the stomach mucosa^3,4^ tablet administrating devices,^5^ spring-like actuators,^6^ and balloon-assisted expanding intestinal delivery devices.^7^ These devices are often fabricated in materials that are bioinert or nonbiodegradable.^3,6,8,9^ There is a need to transition to biodegradable materials for several reasons. First, from the perspective of patient safety. As recent trends in drug delivery have demonstrated usage of more complex oral devices, which differ from traditional oral dosage forms, it is essential to ensure that these devices function and minimize obstruction risk through biodegradability. Second, the impact of large-scale usage and elimination of plastics and microplastics makes it important to ensure that future oral delivery devices do not cause an increase in the generation of nondegradable polymer contamination, especially given the device would enter the sewage system and thus present itself as a potential contaminant.^10^ Third, there could be potential adoption issues, both for the patients and from healthcare systems on a wider scale. From the perspective of patients, there is a possibility of a lack of adoption if the product is known to be fabricated with nondegradable materials. Furthermore, healthcare systems and government agencies may push back on the usage of such devices if these issues of safe disposal and degradation are not addressed.

In previous studies, self-unfolding foils have been fabricated and found to support loading with powder drug formulations.^8,9^ The foils protect the loaded drug and allow for unidirectional release via close proximity to the epithelium, which increases the oral bioavailability when compared to conventional marketed dosage forms. The main elastomer foil material used for producing the devices needs to be safe while providing the elastomeric recovery to enable unfolding at the target location. Elastomeric polymers have also been utilized in other research into oral devices. In a specific case, a luminal unfolding microneedle injector (LUMI) comprising an elastomeric central core with drug loaded arms was demonstrated recently by Abramson et al. The elastomeric core facilitates unfolding in the intestine and injects the microneedle cargo into the epithelial tissue.^6^ A similar unfolding gastroretentive device also uses an elastomeric core to unfold within the stomach for longer term dosing.^11^ In both these cases, the elastomeric core consists of nondegradable thermoplastic elastomers.

The elastic modulus of these elastomeric materials is essential to ensure both the safety as well as the intended functionality of the device during deployment in the gastrointestinal (GI) tract. Previous work by Jørgensen et al. and Ghavami et al. has established the mechanical behavior of an elastomeric foil during folding and subsequent deployment.^8,9^ The dimensions of this device are established based on the physiological dimensions of the targeted intestine. The foil thickness is, to some extent, coupled to the elastic modulus of the foil material. A higher elastic modulus would allow for the reduction of this thickness to produce the same force exerted during unfolding. Keeping the foil thickness low is also important to ensure the full device can be rolled and loaded into a suitable capsule, in this case a size 00 capsule would be used, as this presents the maximum size that is generally considered suitable for swallowing.^12^ To produce a similarly performing device, the replacement elastomer must have an elastic modulus of around 1 MPa. Additionally, the elastomer must support purely elastic deformations at strains ranging up to 40–50%. This requirement is to ensure that the device is not subject to plastic deformation during production or storage. Such irreversible deformation caused by creep or excessive strain would be detrimental to the intended device performance. Thus, finding biodegradable elastomeric materials for oral devices is not a trivial task and requires characterization of the material and consideration of the fabrication techniques needed to produce the final device.

Several biodegradable elastomers have been researched and described over the years including but not limited to polyglycerol sebacate urethane (PGSU),^13^ polyoctane diol citric acid (POC),^14^ and poly(1,3-trimethylene carbonate) (PTMC).^15^ Primarily, these elastomers have been developed and used for tissue engineering applications, however, there has been recent industrial and academic applications in the drug delivery field.^16^ Citric acid-based polyesters, such as POC, offer a large degree of tailorability and facile synthesis. The ratio of reactants, length of the diol chain, curing temperature and time have been demonstrated to affect the final mechanical properties of the elastomer.^17,18^ Additionally, citric acid is a “generally recognized as safe” (GRAS) material and previous studies have demonstrated biocompatibility of the cured elastomer during exposure to cell lines^18^

In this study, we synthesized POC via a one-pot reaction, with subsequent purification and fabrication of a foil-type device comprising a 2D array of microscale compartments that can be used for drug loading. Use of the selected elastomer in a foil-type device was proven to be feasible in the molding process used for defining the microstructures residing on the top surface of the foil. The elastomer and the final device were characterized to demonstrate that the device, based on a biodegradable substitute material, performed in a manner that complied with the oral delivery of macromolecules. Specifically, the chemical composition of the prepolymer was characterized, and the cured elastomer was evaluated via mechanical testing, surface free energy measurements, and degradation studies. This work demonstrates the first usage of POC, an elastomer previously utilized in tissue engineering,^19−21^ for the purpose of oral drug delivery.

## Materials and Methods

### Materials

Citric acid (electrophoresis grade, 99.5+%) was obtained from Thermo Scientific (Waltham, MA, U.S.A.). Octanediol (98% purity), ethanol absolute, sodium hydroxide, phosphate buffered saline, hydrochloric acid, and acetaminophen (98–102% purity) were of analytical grade and obtained from Sigma-Aldrich (St. Louis, MO, U.S.A.). Poly(methyl methacrylate) plates with a thickness of 5 mm and foils with a thickness of 175 μm were purchased from RIAS A/S (Roskilde, Denmark) and Goodfellow (Pittsburgh, PA, U.S.A.), respectively.

### Production and Characterization of the Elastomer

### Synthesis and Purification

**Figure 1:**
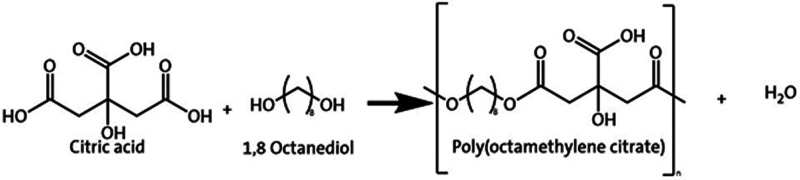
Reaction schematic showing polycondensation reaction between citric acid and 1,8 octanediol.

The prepolymer was synthesized then purified using an adjusted version of the method presented by Koper et al.,^17^ with the reaction schematic shown in Figure 1. Initially, 1,8-octanediol and citric acid in equimolar quantities were reacted under vacuum within a 500 mL round-bottom flask with a stir bar. The reactants were first heated to 160 °C until fully melted; thereafter, the temperature was lowered to 140 °C and left for 1 h. This reaction produced a viscous clear prepolymer. The reaction was halted by placing the round-bottom flask inside an ice bath.

The prepolymer was purified by dissolving it in ethanol (30 wt %). This solution was poured into deionized (DI) water in a volume ratio of 40:150 ethanol/prepolymer:DI water. A white precipitate was formed with a clear viscous polymer sediment. The mixture was centrifuged using 50 mL falcon tubes and spun at 5000 rpm for 10 min. The supernatant was discarded, and the viscous liquid was collected. The viscous liquid was distributed in multiple falcon tubes to reduce the risk of overflow/spillage. The tubes were flash frozen in liquid N_2_ before being freeze-dried overnight (−106 °C, 0.304 mbar). The final product was a viscous clear prepolymer. Samples were stored at room temperature under a vacuum to prevent moisture absorption.

### Chemical Characterization

#### Gel Permeation Chromatography (GPC)

Samples were analyzed using a Shimadzu Prominence fitted with an SPD-M20A photodiode array detector (Shimadzu Europa GmbH, Duisburg, Germany) equipped with a Styragel Mixed D column and a 100% tetrahydrofuran mobile phase run at 1 mL/min. Polystyrene standards of various molecular weights (2050–18100 g/mol) were run to establish a calibration curve. Samples were dissolved in tetrahydrofuran at 5 mg/mL with all samples and standards run by using a 100 μL injection volume. Chromatograms at 280 nm were used to identify elution peaks with corresponding elution times plotted against molecular weight. Subsequently, the prepolymer was run as described above to identify the molecular weight.

#### Nuclear Magnetic Resonance (NMR)

To evaluate the chemical structure of the prepolymer, samples were dissolved in deuterated chloroform (CDCl_3_) and tested for ^1^H and ^13^C NMR using a Bruker AVANCE 400 MHz system (Bruker, Billerica, Massachusetts, U.S.A.).

#### Mechanical Characterization

The mechanical properties of samples where the curing time and temperature were varied were tested using a TA.XTplusC texture analyzer (Stable Micro Systems, Godalming, England). Samples were cut into a “dog bone” shape via CO_2_ laser ablation (Epilog Mini 24, 40 W system, Golden, CO, U.S.A.). The sample dimensions were measured with digital calipers (RS PRO Electronic Digital Caliper DIN862, RS components A/S, Copenhagen, Denmark) and the sample thickness was measured using a micrometer (Insize 3109-25, Insize Europe S.L., Derio, Spain).

Samples were mounted using screw-driven clamp grips, and the texture analyzer was equipped with a 10 kg load cell. Samples were tested by using a pull rate of 500 mm/min. The elastic moduli were obtained by plotting the engineering stress versus strain using OriginPro 2021, with linear fitting used on the linear region of the plot.

#### Degradation Studies

Two sets of degradation studies were carried out, based on previous reports in literature.^18,20^ Identical discs of cured elastomer were made using a biopsy punch (Harris Unicore 6 mm diameter, Sigma-Aldrich, St. Louis, MO, U.S.A.) on a cast sheet of elastomer (thickness 100–200 μm). Two curing times were selected, 3 and 6 days of curing at 80 °C. Additionally three different media were used, 0.1 M NaOH (pH 13), 0.1 M HCl (pH 1), and 1 M PBS (pH 7.4). These media were selected to ascertain accelerated degradation (NaOH) and degradation in physiologically representative media (gastric and distal intestine). The elastomer disc samples were inserted in 15 mL centrifuge tubes and subsequently weighed to determine the starting mass. The tubes were filled with 15 mL of media before being placed in an oven at 37 °C for the desired period of time. Samples were removed, the excess media was removed using a Pasteur pipet, and the discs were rinsed three times using DI water. Samples were then placed into a 50 °C oven for 72 h to ensure the samples were dry. Following this, the samples were weighed within a 15 mL tube.

#### Surface Free Energy and Wettability Measurements

Sheets of cured elastomer were tested to measure the surface free energy (SFE) and water contact angle (WCA) by using a drop shape analyzer (DSA30, KRÜSS GmbH, Hamburg, Germany). The samples were subject to deposition of 2 μL droplets of DI water and diiodomethane. The contact angles of the test liquids were monitored for a duration of 10 min to allow stabilization of the droplet as it was observed that following deposition, the droplets appeared to gradually “wet” the sample surface, which resulted in a temporal decrease in the contact angles for both test liquids. The final 10 measurements were used to extract the final contact angle.

### Design and Production of the POC Elastomer Devices

To recapitulate, the foil-based device consists of a relatively thin (200–700 μm) elastomer foil comprising concave microscale compartments on the top surface, which will interface the epithelium after deployment. The pharmaceutical composition is loaded into said compartments, which are subsequently sealed by deposition of a flexible enteric top layer. Switching from a low surface free energy elastomer like PDMS to the POC elastomer, which has a much higher surface free energy, is advantageous in terms of ensuring good adhesion of the enteric top coating. However, it also makes microscale replication somewhat more difficult.

In the current study, we have focused on devising a replication method in which the prepolymer is cast onto a suitable polymer template with convex microscale protrusions. In order to obtain good pattern replication fidelity, it is paramount that the prepolymer wets the polymer template to ensure complete filling while simultaneously suppressing air inclusion in the casting step. Having a substantial amount of entrapped air bubbles in the relatively viscous prepolymer prior to thermal curing is detrimental in terms of the device integrity and performance. Additionally, the convex topography of the polymer templates should accommodate smooth demolding of the thermally cured devices.

**Figure 2:**
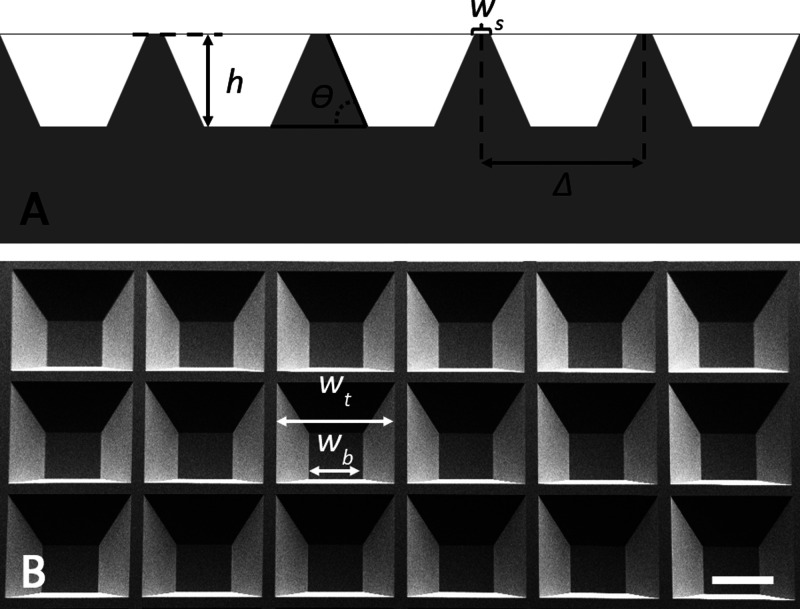
Schematic and SEM image introducing important design parameters. (A) Schematic showing the inverse pyramidal frustum topography of both the silicon master and the final POC elastomer devices. In the schematic, *h* is the compartment depth, Θ is the sidewall taper angle, *w*_s_ is the sidewall width at the top surface, and Δ is the pitch/period of the pyramidal frustums. (B) SEM images of the final silicon master acquired at a tilt angle of 30° to illuminate the topography. The compartment widths at the top, *w*_t_, and bottom, *w*_b_, have been indicated. The scale bar corresponds to 200 μm.

This requirement ultimately motivated a convex topography featuring protrusions with positively tapered sidewalls and a very low surface roughness. The polymer templates were made by hot embossing a fabricated silicon master into thermoplastic polymer foils. The design of the silicon master features a 50 × 50 mm^2^ patterned area comprising a regularly tessellated 2D array of 112 × 112 inverse pyramidal frustums with a pitch of Δ = 447 μm, a compartment depth of *h* = 150 μm, a sidewall taper angle of Θ = 54.7°, and a sidewall width at the top surface of *w*_s_ = 47 μm. A schematic of the design and a representative SEM image of the final silicon master topography are included in Figure 2. As the volume of a single compartment is *V* = (1/3)*h*(*w*_t_^2^ + *w*_t_*w*_b_ + *w*_b_^2^) the overall design volume available for loading pharmaceutical compositions is 169.5 mm^3^ when considering the entire patterned area.

The silicon master was produced using conventional anisotropic potassium hydroxide (KOH) wet etching of a ø100 mm ⟨100⟩ silicon wafer (Siegert Wafers GmbH, Aachen, Germany) having a low stress silicon nitride hard mask with square 400 × 400 μm^2^ openings. The full fabrication details are included in the Supporting Information (Figure S4). The produced silicon master features positively tapered sidewalls and perfectly smooth surfaces and after deposition of an antistick coating it was employed for hot embossing polymer templates used for POC elastomer casting. Different polymer template materials including polycarbonate and cyclic olefin polymer were investigated but ultimately poly(methyl methacrylate) (PMMA, Goodfellow Corporation, Pittsburgh, PA, USA) foils with a glass transition temperature of 105 °C and a thickness of 175 μm were used. Hot embossing was conducted on a desktop imprint system (Compact Nanoimprint Tool, NIL Technology ApS, Kongens Lyngby, Denmark) using a chamber pressure of 20–50 mbar, an embossing temperature of 155–160 °C, an embossing pressure of 6 bar which was maintained for 10 min and a release/demolding temperature of 70 °C. This resulted in perfect inverse replication of the silicon master topography.

#### Casting and Thermal Cross-Linking

Thermal cross-linking was performed at 80 °C, this was selected based on temperature ranges utilized in previous works.^17,20,21^ The samples were cast into polytetrafluoroethylene (PTFE) or acetyl butadiene styrene (ABS) dishes or custom PMMA molds, used to affix PMMA sheets which were hot embossed to provide an inverse relief of the desired microscale topography. The viscosity of the prepolymer used for casting was reduced by dilution using absolute ethanol (15 wt %), to yield a liquid which was suitable for filling syringes. Subsequently, a predetermined volume could be dispensed into the desired molding format. The samples were subject to solvent evaporation and curing in a Binder VD23 vacuum oven (Binder GmbH, Germany). No vacuum was applied during the curing step.

#### Characterization of the Microstructures

The topography of the hot embossed PMMA foils as well as the produced POC elastomer replicas were characterized by vertical scanning interferometry (VSI) using a PLu Neox 3D Optical Profiler (Sensofar Metrology, Terrassa, Spain). Additionally scanning electron microscopy (SEM) was used to image the microstructures.

#### Loading and Coating of the Device

Following curing of POC into the final cured device, samples were loaded manually using acetaminophen powder (Sigma-Aldrich). The foils were weighed before and after API loading to ascertain the loaded mass. The coating formulation and application method used were adopted from Milián-Guimerá et al.^22^ Eudragit FL30D-55 was diluted to 2-wt % in isopropyl alcohol (IPA) prior to lid deposition using ultrasonic spray coating. An ultrasonic spray coater (ExactaCoat system, Sono-Tek, Milton, NY, U.S.A.) equipped with a microbore-fitted 120 Hz Vortex nozzle was used. The coater was programmed to maintain a distance between the tip and the samples of 50 mm, with a total of 800 or 400 passages, as previously described.^23^ During coating, the infusion rate was kept at 0.5 mL/min, and the generator power was kept at 3 W, together with a nozzle translation speed of 50 mm/s. The cladding air pressure and the hot plate temperature were set at 0.04 bar and 40 °C, respectively. Following spray coating, samples were allowed to dry within a fume hood to ensure complete evaporation of IPA.

#### Drug Release

A Pion μDiss Profiler (Pion Inc., Billerica, MA, U.S.A.) was used for determining the release of the model compound (acetaminophen) from the devices. The Pion μDiss Profiler was equipped with online UV probes with 1 mm mirror path lengths, measurement of absorbance taken at 235–250 nm with a sampling frequency of once every 10 s. The dissolution media used was 0.1 M HCl for the first 1 h and the medium was switched to PBS pH 6.8 thereafter. Calibration curves in both sets of media were generated, using stock concentrations of acetaminophen at 78–774 μg/mL. During testing, the initial HCl media was discarded and replaced with the PBS medium. All samples were tested in triplicate at 37 °C.

## Results and Discussion

### Elastomer Properties

#### Chemical Properties of the Prepolymer

As stated previously, the synthesis of the polymer was modified from previous work which used nitrogen flow for removal of water during the reaction.^17,20^ It was decided to react under vacuum with a cold trap to collect the water. This would similarly push the reaction forward through the removal of the undesired byproduct and reduce the risk of hydrolysis of ester bonds.

Based on the characterization conducted via GPC, the weight-average molecular weight of the prepolymer was found to be approximately 3.5 kDa and number-average molecular weight of 0.4 kDa with a multimodal distribution. This is seen in the high PDI of 8. There appears to be three populations of molecular weights in the elugram (Figure S2), a broad peak from a retention time of 8–9 min, a second peak from 9 to 9.5 min, and a third and the largest peak is at 10.15–10.85 min. Figure S2 shows these three peaks, with their molecular weights corresponding to 8.5, 6, and 0.4 kDa, respectively. Previous studies using GPC or mass spectrometry report molecular weights ≤1 kDa.^17,20^ The ^1^H NMR spectra confirmed the chemical structure of the prepolymer with peaks identified from both the citric acid and 1,8-octanediol. Methylene groups from 1,8-octanediol were assigned to peaks at 1.22–1.32, 2.82–2.96, and 3.62–4.74 ppm, and the remaining peak assignments have been detailed in Figure S3. The results indicate that the synthesis of the prepolymer was successful for subsequent casting to make the final elastomeric foil-type devices.

#### Mechanical Properties of Cured Elastomer

**Figure 3:**
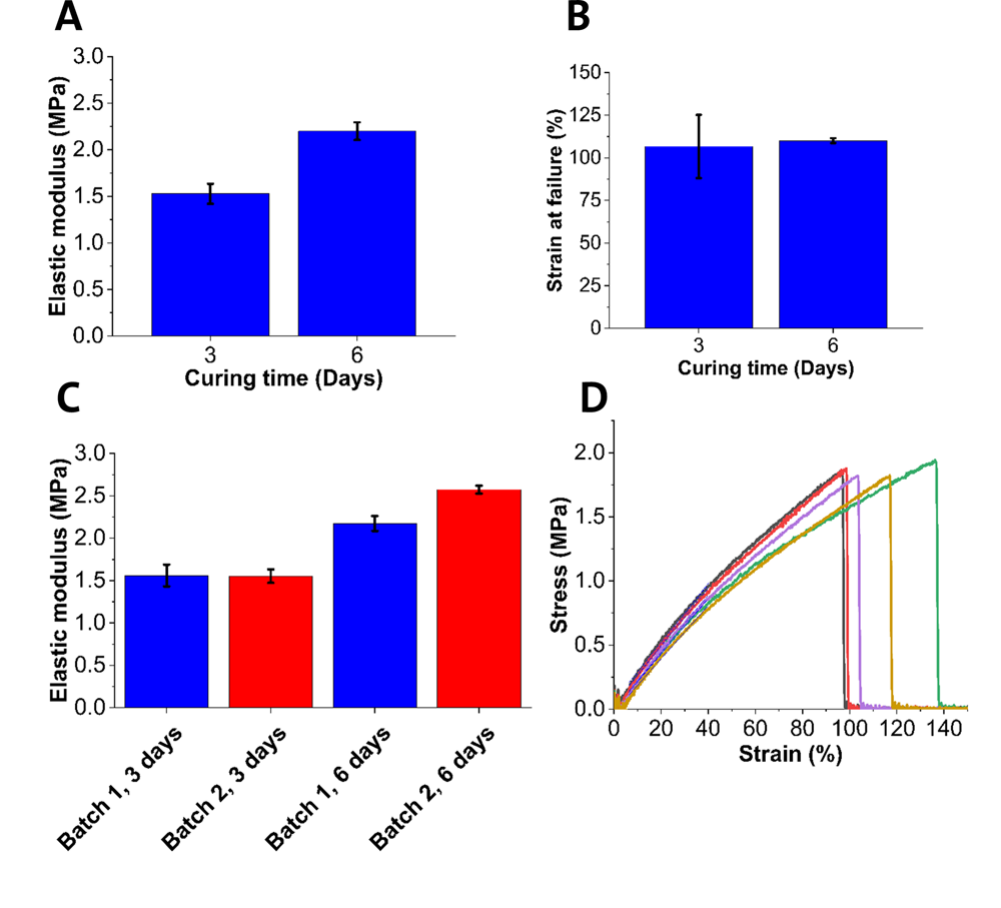
Mechanical properties of the synthesized POC elastomer. (A) Elastic moduli of POC samples were cured for 3 and 6 days. (B) Strain at failure of POC samples was cured for 3 and 6 days. (C) Elastic moduli of POC samples from two separate batches. (D) Engineering stress vs strain graphs for POC samples were cured for 6 days. *n* = 3–6 tested.

Figure 3A shows the mechanical testing of elastomers formed through curing at 80 °C and that additional curing time produced elastomers with a higher elastic modulus. Curing times of 3 and 6 days under these conditions yielded elastic moduli of 1.5 ± 0.1 and 2.2 ± 0.1 MPa, respectively. The measured elastic moduli correspond to those previously reported in literature for similar curing conditions.^17,18^

Both 3 and 6 days cured samples demonstrated elongation at failure at strains greater than 100% (106.6 ± 18.5% and 110.1 ± 1.5% respectively; Figure 3B). Thus, based on previous estimates of the strain conditions,^8^ the cured elastomers would be capable of enduring the rolling process necessary for loading the final device into a capsule. It was also proven that the mechanical properties were consistent across different batches of prepolymer, indicating that the synthesis is robust and reproducible (Figure 3C). Figure 3D shows the engineering stress–strain curves obtained during pull tests on 5 samples, highlighting the linear response up to 40% strain, with the strain at failure above 100%. From these data, we confirmed that under mild curing conditions it is possible to produce POC elastomer having mechanical properties well-suited for device fabrication. Previously conditions as low as 37 °C have been reported to produce POC elastomer, however, it is unlikely that the elastomer produced under these conditions would possess the desired mechanical properties within a reasonable time frame.^20^ It should be noted that given the thermal cross-linking mechanism, further characterization of the kinetics of the cross-linking would be required to ensure the properties of the elastomer during storage are consistent. The elastomer could be kept under cold and low humidity conditions to minimize the risk of further cross-linking/degradation during storage.

#### Degradation Properties

**Figure 4:**
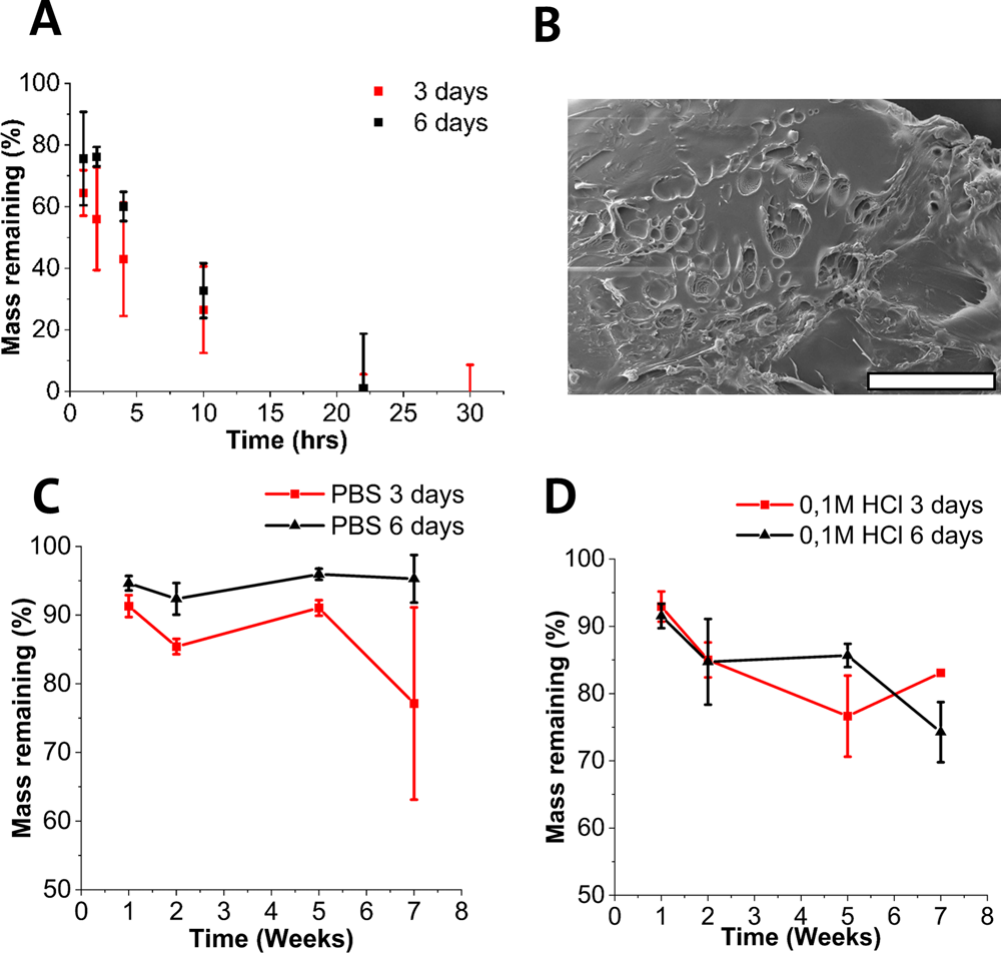
(A) Mass loss over time of POC samples cured for 3 and 6 days when incubated in 0.1 M NaOH at 37 °C. (B) SEM image of a POC 6 day sample after 10 h of incubation. The scale bar corresponds to 500 μm. (C) Mass loss over time of POC samples cured for 3 and 6 days when incubated in 0.1 M HCl (pH 1) at 37 °C. (D) Mass loss over time of POC samples cured for 3 and 6 days when incubated in PBS (pH 7.4) at 37 °C. Each point on the graphs in (A)–(D) corresponds to *n* = 3 measurements.

The accelerated degradation study showed that incubation of samples in 0.1 M NaOH rapidly degraded the elastomer, leading to total degradation after approximately 22 h (Figure 4A). There was no significant difference between 3 and 6 days curing time. This could be attributed to small differences in sample thickness, leading to inconsistencies in sample mass.

Additionally, the rate at which the polymer degrades in 0.1 M NaOH may be too high to meaningfully distinguish between different curing times.

Previous work has shown little difference between similar elastomers when degrading in NaOH, with degradation rates only differing when elastomers were synthesized with longer chain length diols, such as 1,12-dodecanediol.^18,20^ The scanning electron microscopy (SEM) image in Figure 4B shows the surface structure with visible erosion following degradation for 10 h.

POC is typically slowly degrading, with similar studies demonstrating 100% mass loss after 25 weeks.^20^ The elastomers produced in the current study appear to exhibit a reduced degradation rate irrespective of the curing time duration, as seen in Figure 4C,D. There appears to be slight increases in weight between time points within Figure 4C,D. It should be noted that the samples for the degradation samples are not the same set of polymer samples measured over time, but separate polymer samples incubated for each time point. This was done to minimize the impact of drying on the polymer sample during measurement of the sample weight.

**Figure 5:**
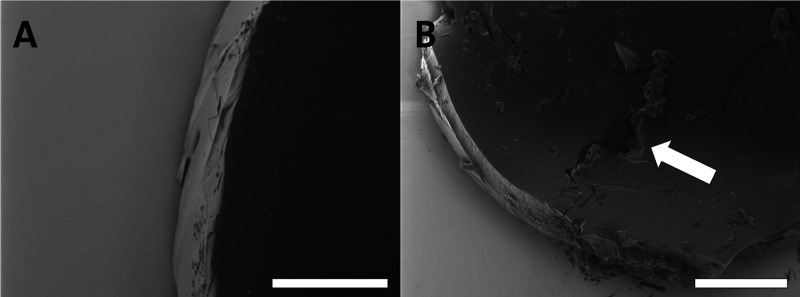
Representative SEM images of POC discs following degradation for 1 week (A) and 5 weeks (B) in HCl with curing times of 6 days. The scale bars correspond to 500 and 1000 μm, respectively.

It is expected that increased cross-linking density, as a result of longer curing times, slows water ingress, thus reducing the rate of degradation. Future characterization of the prepolymer and cured elastomer could use techniques, such as MALDI-TOF, to measure the cross-linking density and molecular weight of samples.^21^ It was observed that, despite the slow mass loss, the morphology of the elastomer changed greatly over time. Between weeks 1 and 5 of the degradation study, it could be seen that the polymer appeared eroded with visible cracks indicated with a white arrow in Figure 5B. The change of the mechanical properties of POC in the wet state has been characterized by Wan et al.^24^ Their work demonstrated a large reduction of greater than 60% in both young’s modulus and elongation at break, for samples incubated in PBS within a period of 5 days.^24^

Based on this behavior, in combination with the observations of our own degradation samples, we believe that such elastomers would likely be excreted following the unfolding and release of the drug content. In this and previous degradation studies, additional parameters such as peristaltic movement and flow of GI fluid have been omitted, which could potentially increase the degradation rate of the elastomer in an *in vivo* setting.

#### Surface Free Energy and Wettability Measurements

**Figure 6:**
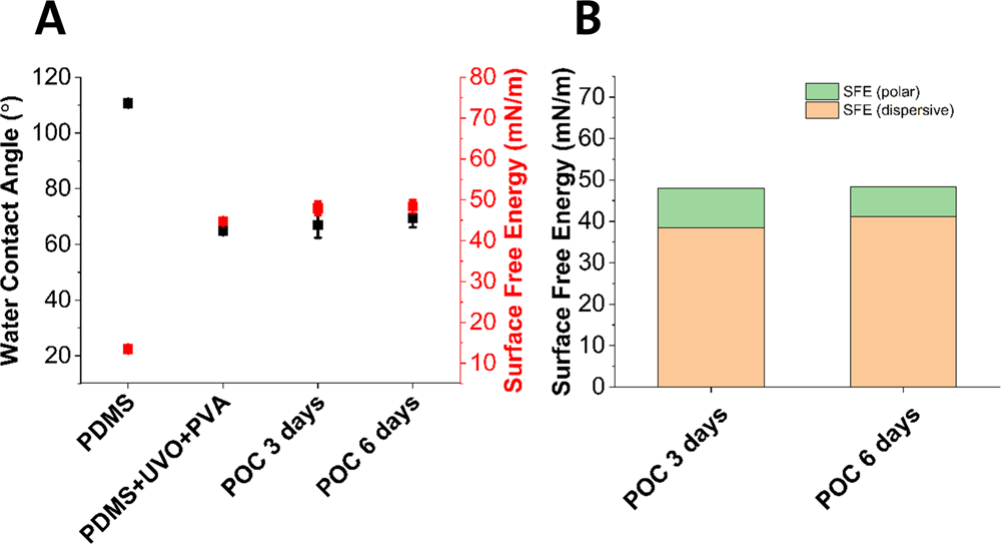
(A) Water contact angles and surface free energy for POC cured for 3 and 6 days compared to those of PDMS with/without UV-ozone treatment and PVA coating. (B) Polar and dispersive contributions to SFE for POC were cured for 3 and 6 days. For each measurement, *n* = 3 samples were tested.

The water contact angle and surface free energy were measured for POC samples cured for 3 and 6 days, and the results were compared to the previous results for PDMS with and without UV oxidation and PVA treatment (Figure 6A).^8^ Additionally, the polar and dispersive parts of the surface free energy were calculated by using the OWRK method (Figure 6B). The hydrophilic nature of POC compared to PDMS can be seen through its smaller initial water contact angle, 65° compared to 110°, respectively. The wettability increase, with respect to PDMS, is highly desired as the POC elastomer requires coating to seal the drug loaded compartments in the foil device. In previous work, this has presented a challenge to successfully adhere functional coatings without UV-ozone and PVA treatment.^8^

It would be expected that the contact angle would increase with the cross-linking density, based on the increase in ester bond formation and subsequent reduction of hydroxyl and carboxyl groups at the surface.^18^ From our measurements, the impact of curing time on the surface free energy appears to be negligible when considering samples cured for 3 and 6 days. This could potentially be attributed to similar cross-linking densities in 3 and 6 day cured samples. This similarity in cross-linking density could also have resulted in similar degradation rates as seen in Figure 4C,D.

During the contact angle measurements on POC, a notable transient decrease was observed over the course of 10 min and the data has been included in Figure S5A–C. Similarly, in Figure S5D, measurements on the transient decrease in droplet volume have been included. This transient behavior is most likely caused by evaporation, potential absorption into the elastomer and ultimately nonidealities in the surface free energy. It goes beyond the scope of the current publication to fully illuminate the temporal wetting behavior of the POC elastomer which would require additional measurements including static and dynamic droplet analysis.

### Foil-Type Devices

#### Microstructure Properties

The microscale cavities into which drugs can be loaded are a key characteristic of the final device. Spin coating was initially evaluated as a potential method to produce foils, comprising concave microscale compartments. Previous work has utilized spin coating of PDMS onto antistick coated microfabricated silicon wafers.^8,9^ During development, we initially attempted to spin coat prepolymer onto planar and microfabricated silicon wafers with antistick coatings. but the prepolymer was observed to dewet the surface while curing. The empirical data has been included in Figure S6. This dewetting phenomenon was more pronounced on antistick coated wafers with microscale features etched into the surface. Subsequently, polymer substrates including polycarbonate and cyclic olefin polymer were tested but here similar wetting issues were evident. (Figure S6C,D). It was hypothesized that as the surface free energy is increased, the prepolymer wettability increases, which promotes formation of a continuous POC elastomer film. The FDTS coated wafer, cyclic olefin polymer, polycarbonate and PMMA, represent a series of materials having increasing surface free energies which could explain the observed improvement in the formation of a continuous POC prepolymer layer.^25,26^ Additionally, the patterning of the surface may alter the surface free energy, leading to wetting in patterned and dewetting in nonpatterned regions (Figure S6D).

**Figure 7:**
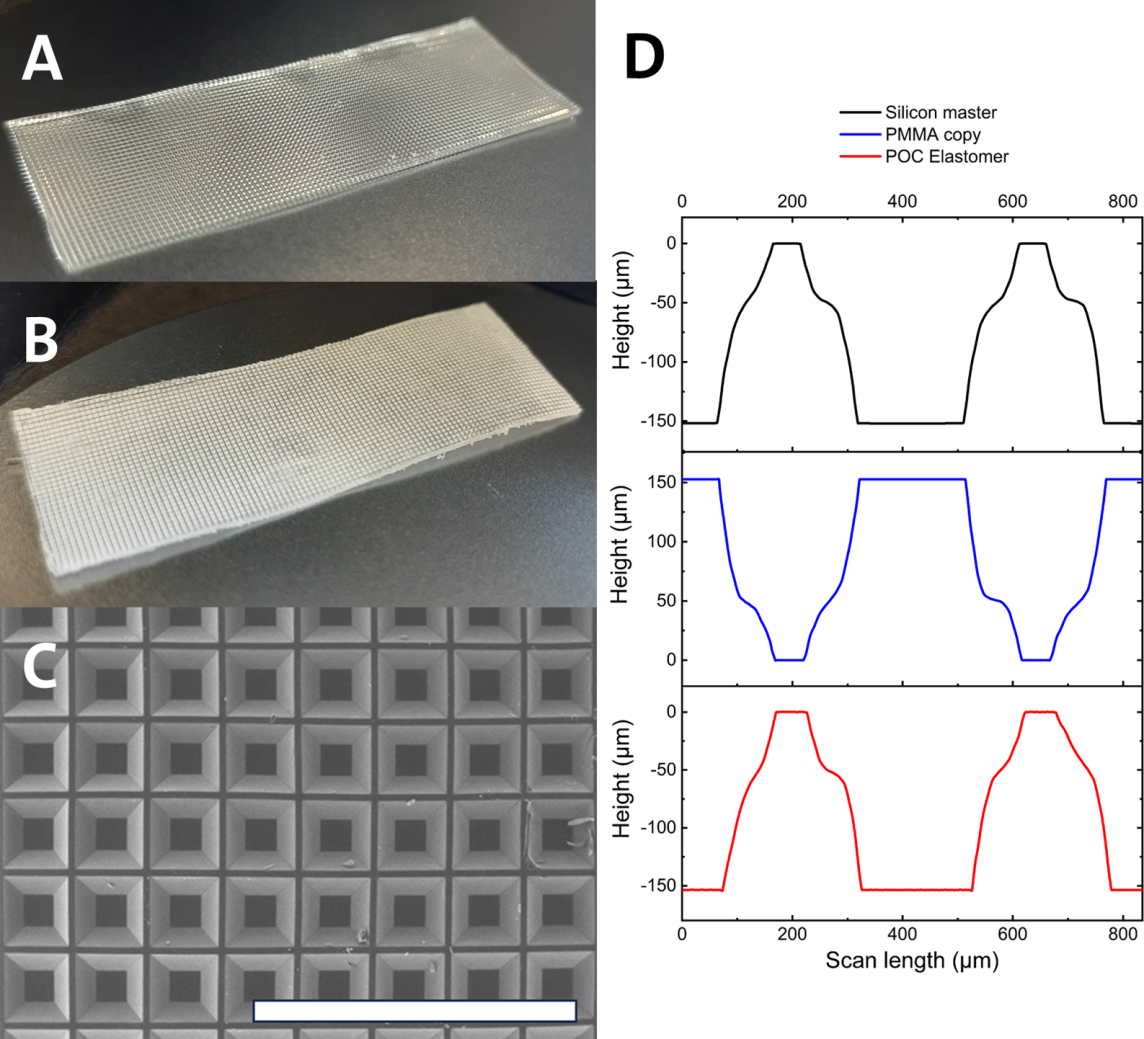
(A) Picture of the full unloaded device. (B) Picture of the full loaded and coated device. Both device dimensions are 50 mm × 15 mm × 700 μm. (C) SEM image of the final POC elastomer showing the 2D array of microscale concave compartments. The scale bar corresponds to 2 mm. (D) Cross-sectional profiles from VSI measurements on the silicon master, the PMMA replication, and the final POC elastomer. Based on *n* = 5 measurements (center and the four corners of the patterned 50 × 50 mm^2^ area) the compartment depths on the silicon master and the POC elastomer is 152.9 ± 0.9 and 151.5 ± 0.3 μm, respectively.

The usage of PMMA substrates additionally allowed the creation of molds which were suitable for casting the prepolymer, as they could additionally undergo oven curing to produce the final cured elastomer. As mentioned previously, the design and final topography of the silicon master should alleviate release of polymer templates after hot embossing as well as POC elastomer release from said polymer templates after casting and thermal curing of the prepolymer. Optical profilometry and SEM demonstrated the successful replication of inverse pyramidal frustums in the final cured elastomer (Figure 7C,D). These foil-type devices have two key characteristics to enable their function: elastic recovery and feasibility for the molding of microstructures.

POC could be utilized in other devices, provided that the critical attributes are defined and consistent with the properties of this elastomer. POC could be utilized to form spring-like or energy storing features within other device designs.

#### Drug Release from the Devices

**Figure 8:**
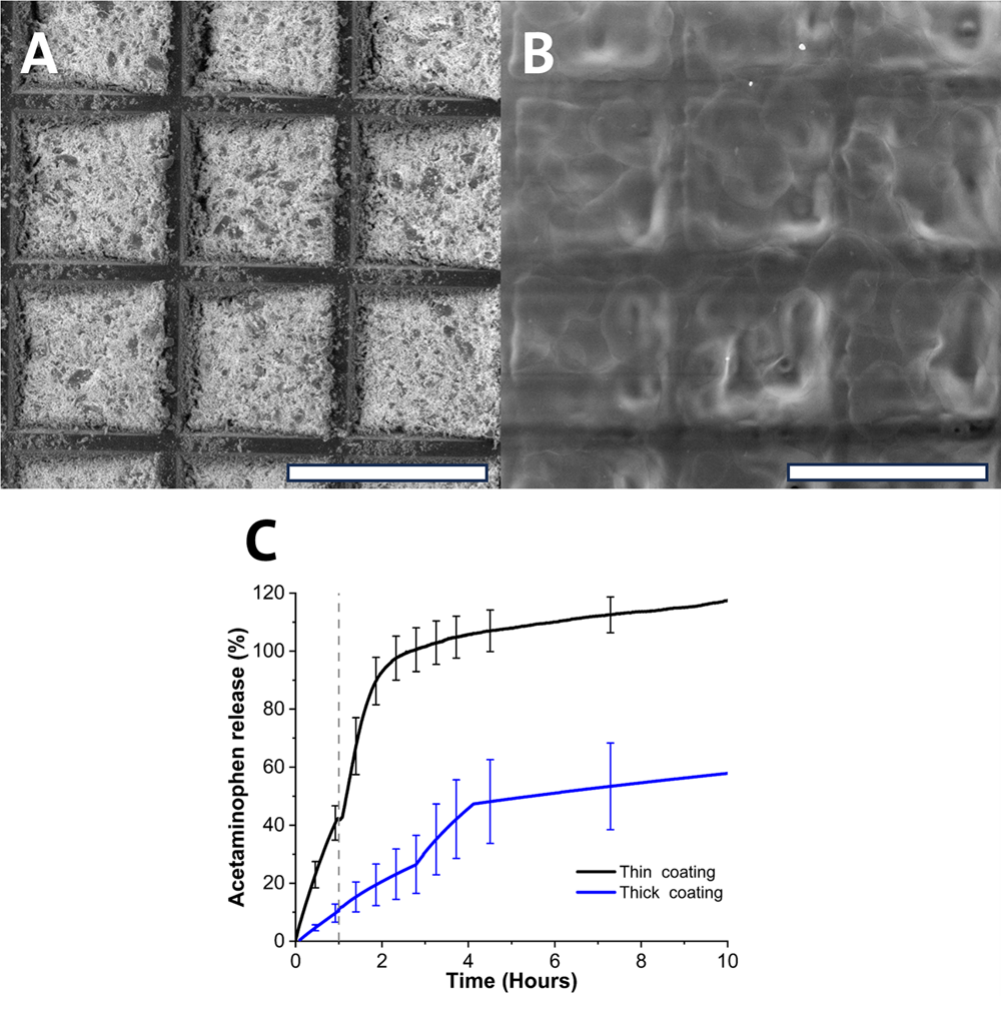
(A) SEM image of an acetaminophen loaded foil without a top coating. The scale bar corresponds to 500 μm. (B) SEM image of acetaminophen foil with a Eudragit FL30D-55 top coating. The scale bar corresponds to 500 μm. (C) Dissolution profiles showing the release of acetaminophen from foils having a thin and thick top coating (c.26 and 13 μm thickness). Gray dashed line denotes change of media from 0.1 M HCl to PBS pH 6.8. Measurements were carried out on *n* = 3 samples.

Dissolution tests were performed for 1 h in 0.1 M HCl (pH 1), followed by pH 6.8 PBS (Figure 8C). The release of acetaminophen from devices coated with thin or thick layers of enteric coating was investigated.

The coating formulation and procedure used have previously yielded coatings with thickness of 26.63 ± 0.77 μm when 400 loops of coating were applied denoted above as “thick”. FL30D-55 consists of a combination of an enteric and flexible coating, yielding a flexible film suitable for application to these devices.^22^ Subsequently, 400 and 200 loops were selected to assess the impact of coating thickness on acetaminophen release profiles.

The POC elastomer of the device has a much slower degradation rate, as observed in Figure 4C,D, this would mean the coating type and thickness would dictate the release kinetics of the drug cargo.

Both release profiles show a gradual release followed by plateau, with the thin coating in Figure 8C demonstrating a preferable quicker release rate based on transit within the stomach and small intestine.^27^ It is likely that during coating, some acetaminophen is associated within the top coating, Figure Figure 8C shows greater than 10% release during this gastric phase (200 loops sample), which would not classify as enteric according to United States pharmacopoeia, however these devices will be administered by capsule.^28^ Subsequently, it is appropriate to use both the enteric coating of the foil-type device, for protection prior to unfolding, with the primary enteric protection from the enteric capsule into which the device is loaded. Capsules were not explored within this work as the usage of enteric capsule was successfully shown in previous studies.^8,9^

## Conclusions

POC demonstrated its capability in replacing existing nondegradable materials, while presenting additional considerations for fabrication. Alteration of the mold design enables molding of the prepolymer to fabricate the final elastomer into the foil-type device. The mechanical properties of the elastomer can be altered through adjusting curing conditions, with the desired elastic modulus and elongation at failure demonstrated in synthesized elastomers. Changing the material used enables production of more sustainable oral devices that are safe for both the patient as the end-user of the oral device and the environment.

Future work should investigate loading the device with macromolecule drugs; currently, only acetaminophen has been demonstrated for its loading and release within the devices. This is convenient for cost reduction and screening purposes; however, it would be interesting to assess the release behavior of more complex and higher molecular weights molecules. Additionally, heat-sensitive drugs such as Semaglutide should be able to load into the device. This is possible as the device is subjected only to temperatures up to 40 °C during the coating process.

The fabrication of an oral device from POC presents the capability to replace nondegradable elastomers, which have been key features in previous research into oral devices.^6,8,9^ Moving forward, it would be interesting to see the continued development and usage of biodegradable materials within oral devices to reduce their potential environmental impact and minimize any risks of obstruction.

## Supporting Information Available

Figure S1: GPC calibration curve; Figure S2: Retention time of prepolymer sample; Figure S3: ^1^H NMR of prepolymer sample; Figure S4: Silicon master production method; Figure S5: Additional WCA and SFE data of POC samples; Figure S6: Casting substrate screening (PDF)

## Abbreviations

ABS: Acetyl butadiene styrene
API: Active pharmaceutical ingredient
DI: Deionized
GI: Gastrointestinal
GPC: Gel permeation chromatography
GRAS: Generally recognized as safe
HMDS: Hexamethyldisilazane
IPA: Isopropyl alcohol
LPCVD: Low pressure chemical vapor deposition
NMR: Nuclear magnetic resonance
PBS: Phosphate buffer saline
PDMS: Polydimethyl silane
PMMA: Polymethyl methacrylate
POC: Polyoctanediol citrate
PTFE: Polytetrafluorethylene
PTMC: Poly(1,3-trimethylene carbonate)
PVA: Polyvinyl alcohol
SEM: Scanning electron microscopy
SFE: Surface free energy
WCA: Water contact angle
UV: Ultraviolet
VSI: Vertical scanning interferometry
