# Supplementary material for: Fully Biodegradable Elastomer-Based Device for Oral Macromolecule Delivery
Source: ACS Appl Bio Mater. 2024 May 16;7(6):3777–85. doi: 10.1021/acsabm.4c00147 (PMC11186471; doi:10.1021/acsabm.4c00147)
Supplement: Supplementary file 1 — mt4c00147_si_001.pdf [file mt4c00147_si_001.pdf]

Supporting information

Fully biodegradable elastomer-based device for oral macromolecule delivery

Reece McCabe*, Lasse Højlund Eklund Thamdrup, Mahdi Ghavami, Anja Boisen*

Corresponding authors

Reece McCabe: reemc@dtu.dk

Professor Anja Boisen: aboi@dtu.dk

The Danish National Research Foundation and Villum Foundation's Center for Intelligent Drug Delivery and Sensing Using Microcontainers and Nanomechanics (IDUN), Department of Health Technology, Technical University of Denmark, 2800 Kgs. Lyngby, Denmark.

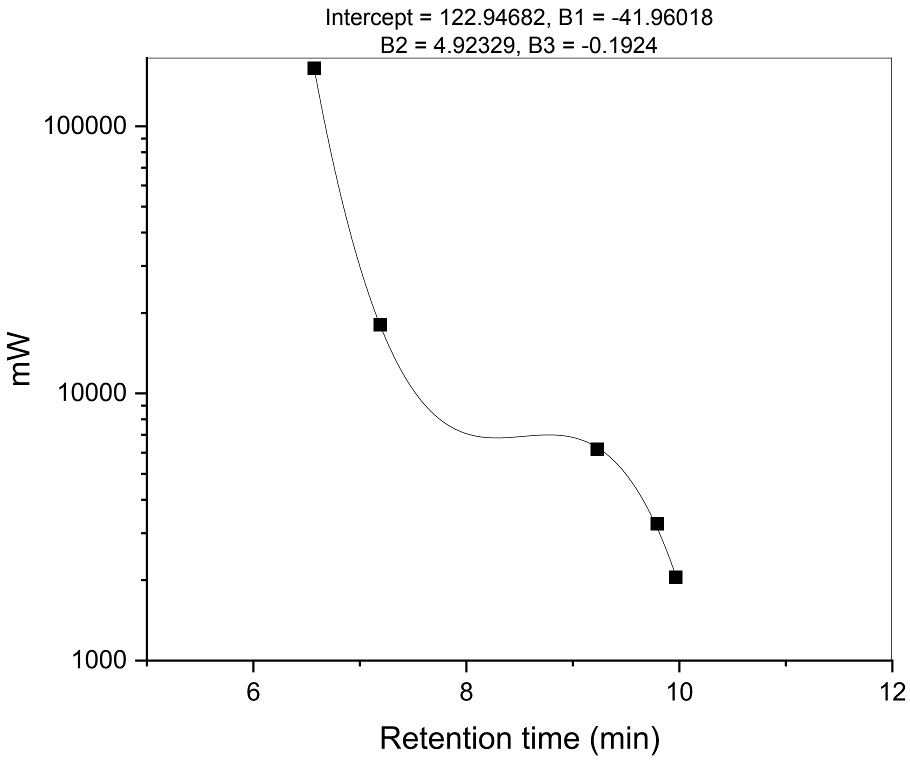

Figure S1: GPC calibration curve of polystyrene standards with respective retention time

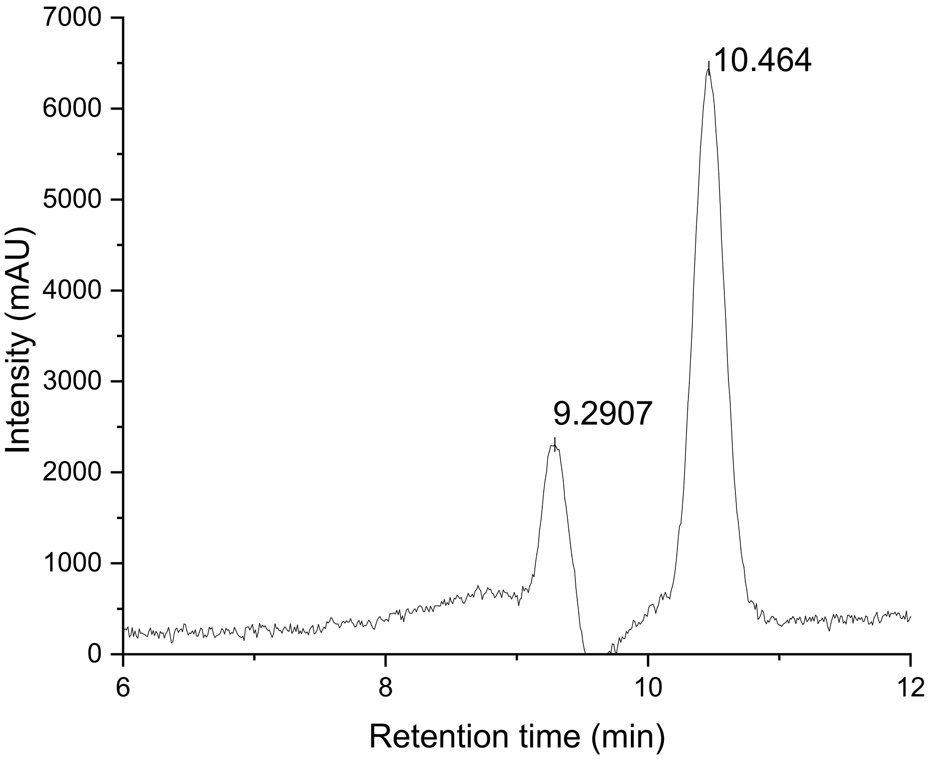

Figure S2: Retention time of prepolymer versus intensity at 280 nm

Poly (1,8 octanediol-co-citrate) 1,8 Octanediol denoted as OD, citric acid denoted as CA.

^{1}H NMR (400 MHz, $CDCl_3$, ppm) 3.74-3.62 ppm (-O$CH_2CH_2(CH_2)_4$-, *from* OD), 2.96-2.82 (-$CH_2)_4CH_2CH_2$O-, *from* OD), 1.61 (-OCO-$CH_2C(OH)CH_2$-OCO-, *from* CA), 1.32-1.22 (-O$CH_2(CH_2)_6CH_2$O-, *from* OD).

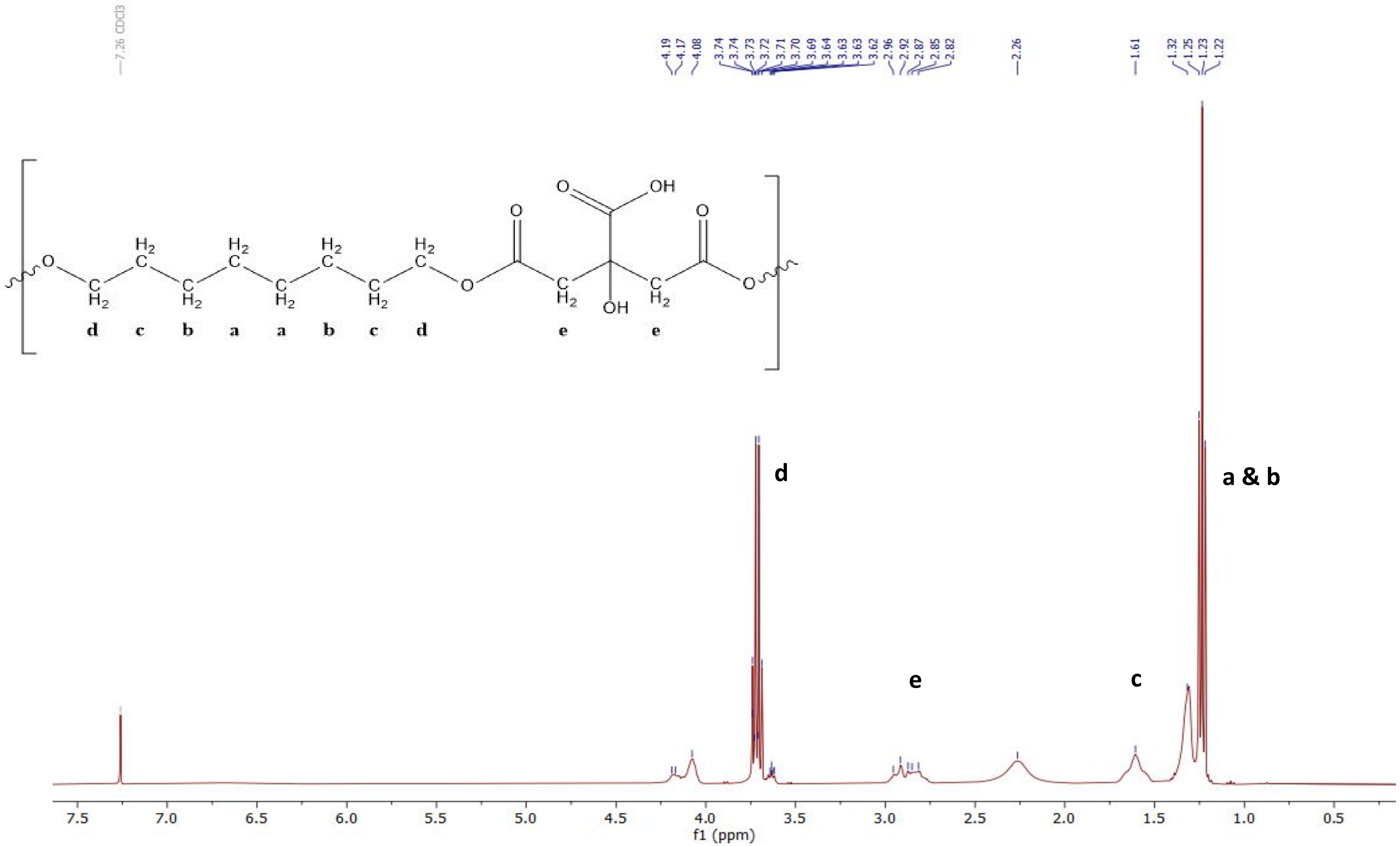

Figure S3: 1H NMR of prepolymer

Detailed description of the silicon master production

For production of silicon masters, ø100 mm single-side polished <100> Si substrates (Siegert Wafers GmbH, Aachen, Germany) with a thickness of 525 µm were used. The Si substrates were initially subject to low pressure chemical vapor deposition (LPCVD, Tempress, Vaassen, The Netherlands) of an approximately 220 nm thick low stress silicon nitride which serves as an effective hard mask during the anisotropic potassium hydroxide (KOH) etch. After the silicon nitride deposition, the substrates where subject to hexamethyldisilazane (HMDS) treatment followed by spin coating (Süss MicroTec Gamma 4M spin coater, Süss Microtec, Garching, Germany) of 1.5 µm thick positive AZ5214E photoresist (MicroChemicals GmbH, Ulm, Germany). The resist was UV exposed using a maskless aligner (MLA150 Maskless Aligner, Heidelberg Instruments, Heidelberg Germany) operating with a laser wavelength of 405 nm. The utilized dose was 80 mJ/cm^2 and the exposed resist was developed in AZ®726 MIF (MicroChemicals GmbH, Ulm, Germany) for 90 s using a single-puddle approach (Süss MicroTec Gamma 2M developer, Garchen, Germany). After development, the resulting resist pattern was transferred to the silicon nitride layer using an Advanced Oxide Etcher (AOE, STS MESC Multiplex ICP, SPTS Technologies Limited, Newport, United Kingdom) with 5 sccm C_4F_8, 4 sccm H_2 and 174 sccm He as the reactive gasses. The chamber pressure during etch was 4 mTorr, the coil|platen power was set to 1300 W|200 W and the platen temperature was 0°C. After the silicon nitride dry etch, the remaining resist was stripped using high energy oxygen plasma in a barrel asher (300 Semi Auto Plasma Processor, PVA Tepla AG, Wettenberg, Germany) and submersion into 7-up at 80°C. The 7-up consists of concentrated H_2SO_4 with $(NH_4)_2S_2O_8$ salt added just prior to immersion into the solution that effectively strips any remaining traces of resist. The anisotropic KOH wet etch was conducted in a dedicated bath kept at 80°C with automatic DI water dosing to keep the KOH concentration steady at 28 wt% throughout the anisotropic wet etch. The etch was terminated when the depth of the pyramidal frustum compartments was approximately 150 µm. Hereafter, the remaining silicon nitride mask was stripped in concentrated phosphoric acid at 160°C by immersion for 4 hours. To remove potential contamination on the substrates, they were subject to a final 7-up clean before depositing an anti-stick coating by molecular vapor deposition (MVD 100 Molecular Vapor Deposition System, Applied Microstructures Inc., San Jose, CA, USA). The MVD process ensures a highly conformal deposition of 1H,1H,2H,2H-perflourodecyltrichlorosilane (FDTS) which effectively lowers the surface free energy of the produced silicon masters thereby facilitating easy demolding of polymer foils after hot embossing. The topography of the produced silicon substrates was characterized and inspected using a combination of optical microscopy (Nikon ECLIPSE L200, Tokyo, Japan), vertical scanning interferometry (VSI, PLu Neox 3D Optical Profiler, Sensofar Metrology, Terrassa, Spain) and scanning electron microscopy (SEM, Zeiss Supra 40 VP, Jena, Germany). The characterization has been summarized in Figure S4. From the picture of one of the final silicon masters, it is evident that it features a central 50x50 mm^2 patterned area comprising a 2D array of 112x112 pyramidal frustum compartments where the width of the tapered sidewalls is approximately 44 µm at the top surface. The sidewall taper angle is dictated by the angle between the <100> and <111> crystal planes which is approximately 54.7°. Based on VSI measurements in the center and the four corners of the patterned area, the average depth of the compartments was 152.9±0.9 µm.

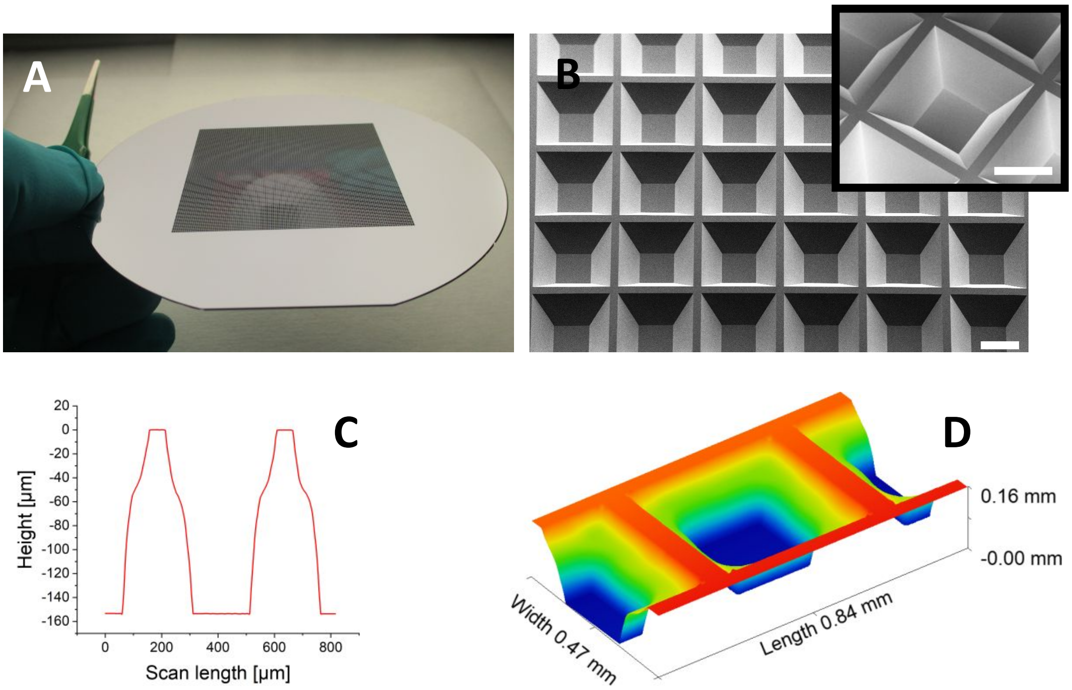

Figure S4: Characterization of the silicon master used for making polymer replicas for POC elastomer casting. **A** Picture of one of the ø100 mm silicon masters showing the 50x50 mm² patterned area consisting of concave pyramidal frustums. Note the camera reflection which is caused by the tapered sidewalls. **B** SEM images of the final silicon master. The compartments have been etched to a depth of 152.9±0.9 µm and the inter-compartment spacing is approximately 44 µm. The sidewall taper angle is approximately 54.7°. The scale bars correspond to 200 µm on both images. **C** and **D** Cross-sectional profile and 3D topography map based on VSI measurements on one of the final Si masters.

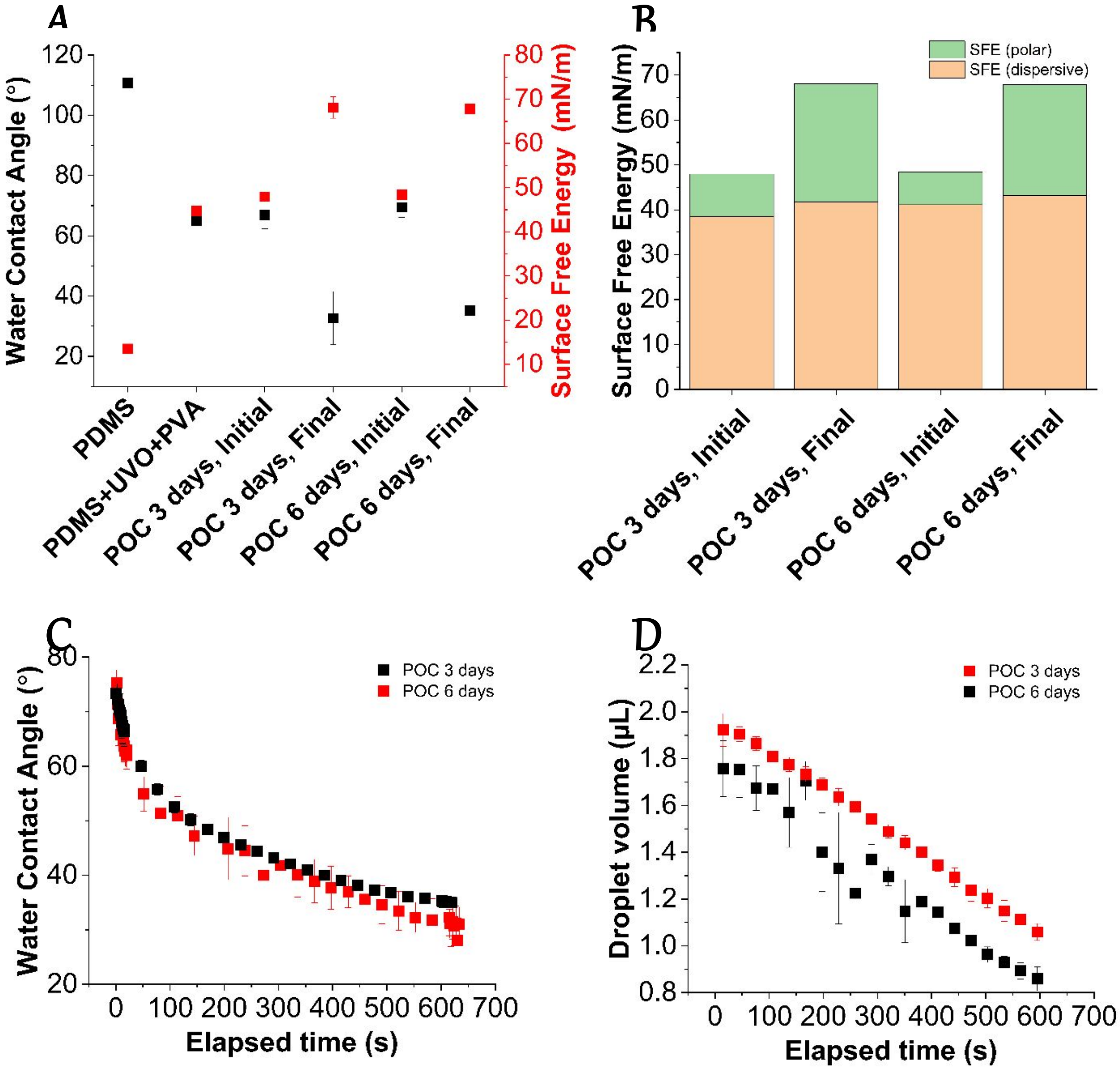

Figure S5: **A** Initial and final water contact angles and surface free energy for POC cured for 3 and 6 days compared to PDMS with/without UV-ozone treatment and PVA coating. **B** Polar and dispersive contributions to SFE for initial and final measurements on POC samples. **C** Reduction of contact angle over time during measurements on POC samples. **D** Reduction of droplet volume over time during measurements on POC samples.

Casting substrate screening

Previously etched FDTS coated silicon wafers were used to spin coat PDMS as a thin layer, subsequently the PDMS could be removed yielding patterned foils. The same technique was tried by casting pre-polymer diluted in ethanol (15wt% ethanol) onto silicon wafers, followed by spin coating. The same process was also tested using different polymers, where both plain and patterned polymer sheets, produced via hot embossing, were trialed. As seen below the different materials demonstrated various wetting issues, meaning that thin films could not be formed. Finally, PMMA was identified as a suitable material which could be hot embossed with the desired pattern, whilst not presenting the wetting issues seen below.

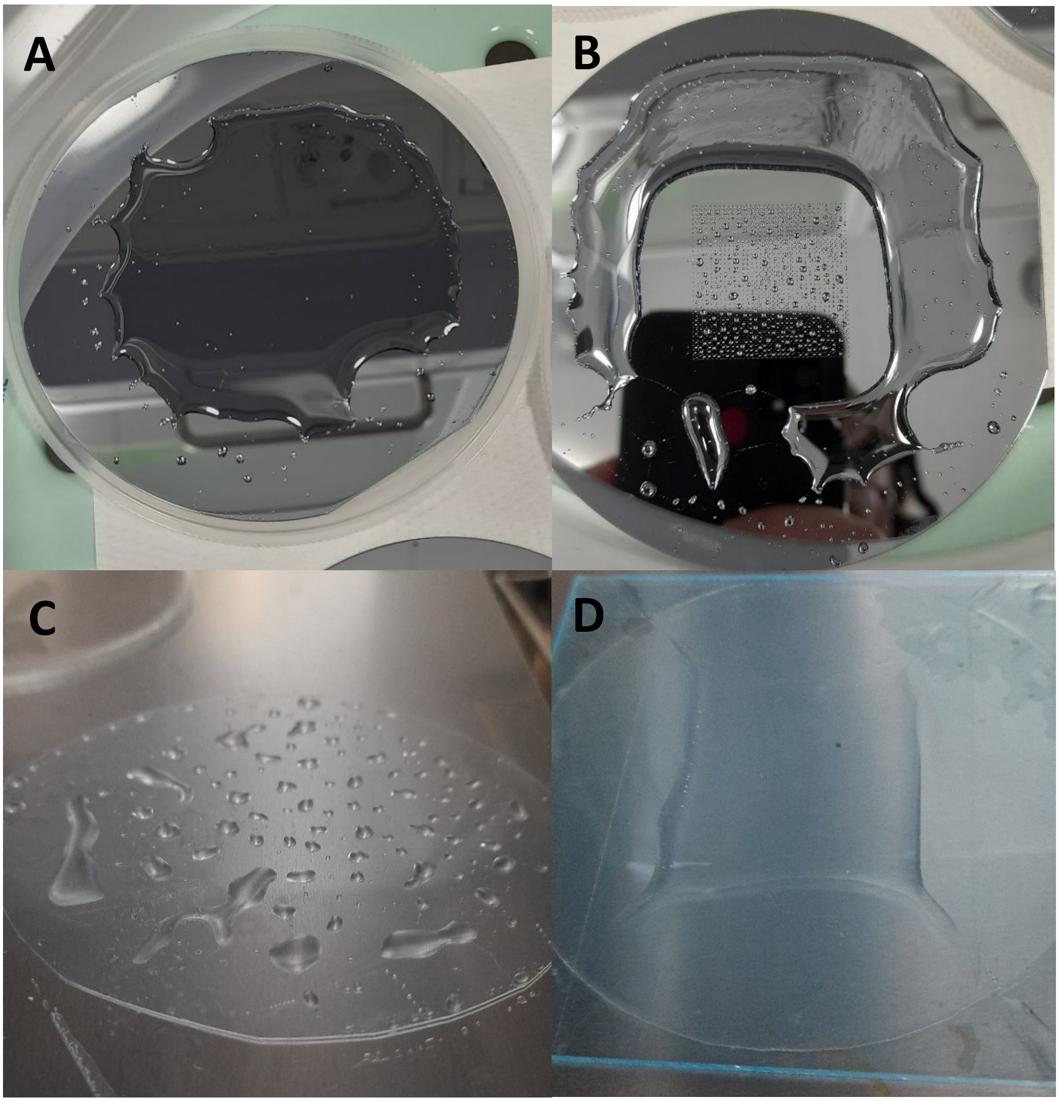

Figure S6: Preliminary attempts of spin coating using various substrate materials including **A** planar FDTS coated silicon wafer, **B** Etched silicon wafer with FDTS coating **C** polycarbonate foil **D** and cyclic olefin polymer foil. All materials were cast to form a thin layer onto a ø100 mm diameter disk and during curing, the pre-polymer could be seen to “de-wet”, causing a patchy appearance and subsequently not forming a usable cured

polymer. In **D,** *the POC elastomer can be seen to only adhere to the central patterned area, with similar lack of coverage on the remaining surface area of the substrate. All images above show the appearance after curing at 80°C without vacuum for 3 days.*
